# Chronic kidney disease is a key predictive factor for potential myocardial ischaemia and poor prognosis in asymptomatic patients with diabetes mellitus

**DOI:** 10.1038/s41598-022-14472-8

**Published:** 2022-06-17

**Authors:** Yuki Honda, Kohei Wakabayashi, Chisato Sato, Naoko Ikeda, Ken Sato, Toshiaki Suzuki, Keita Shibata, Toshiro Shinke

**Affiliations:** 1 Division of Cardiology, Department of Internal Medicine, Fuji Hospital, 1784, Niihashi, Gotemba, Shizuoka 412-0043 Japan; 2 Division of Cardiology, Cardiovascular Centre, Showa University Koto-Toyosu Hospital, 5-1-38, Toyosu, Koto-ku, Tokyo, 135-8577 Japan; 3 Division of Diabetes and Metabolism, Department of Internal Medicine, Fuji Hospital, 1784, Niihashi, Gotemba, Shizuoka 412-0043 Japan; 4 Division of Cardiology, Showa University Hospital, 1-5-8, Hatanodai, Shinagawa-ku, Tokyo, Japan

**Keywords:** Cardiology, Cardiovascular diseases, Diabetes, Kidney diseases

## Abstract

Some asymptomatic patients with diabetes mellitus (DM) have critical coronary artery disease (CAD), although the guidelines do not recommend aggressive screening for CAD in asymptomatic patients. Chronic kidney disease (CKD) is among the serious co-morbidities of severe systemic atherosclerosis. Thus, CKD may be associated with potential myocardial ischaemia. Therefore, the present study aimed to determine the impact of CKD on the incidence of silent myocardial ischaemia (SMI) and the long-term outcomes in asymptomatic patients with DM. This study investigated 461 consecutive patients with DM. All patients who were asymptomatic and self-sufficient in daily life underwent the ergometer exercise (ERG) test. Coronary angiography was performed if the stress test was positive, or if the patient did not achieve 90% of the target heart rate. The primary end point included major adverse cardiac and cerebrovascular events (MACCE) including death, non-fatal myocardial infarction and stroke. The median follow-up duration after study enrolment was 35 months for the entire cohort of 461 patients. Eighty-one patients were diagnosed with SMI. The estimated glomerular filtration rate was significantly lower in the SMI group (70.5 ± 23.8 vs. 81.8 ± 30.0 mL/min/1.73 m^2^, *P* < 0.001). SMI occurred more frequently in patients with advanced CKD [27/103, (26.2%) in stages 3–5], whereas only 5/68 (7.3%) patients without CKD, 13/81 (16.0%) patients with stage 1 CKD and 36/209, (17.2%) in stage 2, had SMI. The Kaplan–Meier curves revealed that, patients with SMI had poor clinical outcomes (log-rank: *P* = 0.016). The incidence of MACCE (log-rank: *P* = 0.009) was higher in patients with severe CKD > stage 3a in the SMI subgroup. Urinary albumin (mg/gCr) was associated with MACCE in the SMI subgroup [HR 3.37, 95%CI (1.170–9.521), *P* = 0.025] after adjusting for age, sex, and conventional risk factors. SMI was more prevalent in patients with CKD and the incidence was proportional to the CKD stage in asymptomatic patients with DM. Those Patients with CKD and SMI exhibited poor clinical outcomes. CKD may be a key factor for the identification and management of SMI in asymptomatic patients with DM in routine clinical practice.

**Trial Registration:** UMIN000038340.

## Introduction

Coronary artery disease (CAD) is a major cause of morbidity and mortality especially in patients with diabetes. Previous studies have demonstrated that patients with diabetes generally carry two-fold elevated risk of CAD^1^. Furthermore, silent myocardial ischaemia (SMI) is more frequently observed in the diabetic population compared to the non-diabetic population^2,3^. However, it is difficult to detect potential CAD in asymptomatic patients with diabetes in routine clinical practice. Thus, delayed diagnosis of ischaemic heart disease could be associated with more extensive coronary atherosclerosis and exacerbation of the outcomes in these patients. On the other hand, the guidelines do not recommend routine examination for CAD using non-invasive modalities such as computed tomography coronary angiography (CTCA) or myocardial perfusion single-photon emission CT for all asymptomatic patients with diabetes, because numerous patients with diabetes do not have potential CAD, and the incidence of cardiovascular events is not extremely high in the overall population of asymptomatic patients with diabetes^4,5^. This results in a dilemma and a conflict between two different perspectives. The early diagnosis of CAD and optimal treatment according to the guidelines are crucial in specific susceptible populations; however, there are no guidelines for the identification of very high-risk asymptomatic patients with diabetes.

It is, thus, unknown how we should find the SMI and what is the key factor for the incidence of SMI and long-term outcomes. Such information is needed to find potential high-risk patients and treat them with the optimal therapy in our daily clinical practice for asymptomatic patients with diabetes. Chronic kidney disease (CKD) is among the most serious co-morbidities associated with diabetes. The reduction in the estimated glomerular filtration rate (eGFR) or the development of albuminuria is known to be associated with cardiovascular disease and mortality^6^. Thus, CKD may be a pivotal factor for the prediction of potential myocardial ischaemia and poor clinical outcomes in asymptomatic patients with diabetes mellitus (DM). However, patients with diabetes and co-morbid moderate to severe CKD are likely to be followed-up intentionally without examination for SMI in clinical practice. Examinations that require the administration of contrast media, such as CTCA or coronary artery angiography, are often avoided due to concerns regarding contrast-associated acute kidney injury, even though systemic atherosclerosis is associated with renal dysfunction. We hypothesized that the severity of CKD would be associated with the presence of SMI and poor clinical outcomes in asymptomatic patients with DM. Therefore, the present study aimed to determine the impact of CKD on the incidence of SMI and long-term outcomes in asymptomatic patients with DM.

## Methods

### Study population

The present study utilized data from a prospective registry of type 2 DM without symptoms (UMIN000038340), which was started in July 2011. The data was retrospectively analyzed for the purpose of the present study. The registry enrolled consecutive patients with type 2 DM who were asymptomatic and self-sufficient with the activities of daily living. Patients with type 1 DM were not enrolled. Symptomatic patients and/or those who were unable to perform the ergometer exercise (ERG) test were also excluded from the registry. Outpatients who visited the diabetes department at Fuji Hospital were prospectively enrolled in the registry. The diagnosis of DM was based on the American Diabetes Association criteria^7^.

### Study design and protocol

According to the protocol of the registry, all patients underwent a cycle ERG test. Twelve lead electrocardiography (ECG) was performed continuously for monitoring during exercise and recovery for 10 min. The blood pressure was recorded at rest and at 1-min intervals during the test. The criteria for a positive stress test were as follows: > 1 mm down-sloping or horizontal ST-segment depression from baseline occurring 60 ms after the J point, accompanied by > 2 mm depression if the resting ECG already demonstrated ST-segment depression, or > 1 mm ST-segment elevation^8^. The stress test was considered inconclusive if the patient failed to achieve 90% of the age-predicted target heart rate. Coronary angiography was performed for patients with positive or inconclusive ERG test. Lesions with > 50% narrowing of the luminal diameter were designated as significant. The diagnosis of SMI based on the exercise stress test results and detection of a significant stenotic lesion. Fractional flow reserve was used to support the diagnosis of SMI based on the physician’s discretion. Those diagnosed with SMI were treated with medication only and/or revascularisation with percutaneous coronary intervention or coronary artery bypass graft surgery. The treatment strategy was left to the discretion of the cardiology team, which consisted of two interventional cardiologists, two general cardiologists and two surgeons. The primary endpoint included a composite of major adverse cardiac and cerebrovascular events (MACCE), including all-cause death, non-fatal myocardial infarction, and stroke.

All participants provided written informed consent before enrolment in the present study, which was conducted in accordance with the Declaration of Helsinki and approved by the local ethics committee of Fuji Hospital.

### Definition and data collection

Laboratory tests, chest radiography, 12-lead ECG, and transthoracic echocardiography were performed during study enrolment. The laboratory tests included haemoglobin A1c (HbA1c) levels, eGFR, brain natriuretic peptide (BNP) levels, and urinary albumin levels. Condition pertaining to the medical history were defined as follows. Hypertension was defined as blood pressure ≥ 140/90 mmHg or treatment with antihypertensive drugs. Dyslipidaemia was defined as a low-density lipoprotein level ≥ 140 mg/dL, high-density lipoprotein level ≤ 40 mg/dL, triglyceride level ≥ 150 mg/dL, or treatment with medication. The disease duration of DM was defined as the interval between the first diagnosis of DM and enrolment in the present study. eGFR was calculated using revised modification of diet in renal disease (MDRD) equations for Japanese. According to previously validated calculations [reference], eGFR (ml/min/1.73 m^2^) = 194 × Serum creatinine − 1.094 × Age − 0.287 × 0.739 (if female)^9^. CKD was defined as the presence of albuminuria of more than 30 mg/g creatinine and/or the reduction in the eGFR of less than 60 mL/min/1.73 m^2^. Diabetic retinopathy was defined as the appearance of progressive dysfunction of the retinal vasculature secondary to chronic hyperglycaemia. An ophthalmologist conducted a screening examination for each patient.

Acute myocardial infarction was diagnosed based on general universal definitions^10^. Stroke was diagnosed by collating the findings of neurological examination and magnetic resonance imaging. The participants were followed up at our institute. Those who could not be followed up at our hospital were questioned by telephone.

### Statistical analysis

The categorical variables were summarized as frequencies and percentages and between-groups comparisons were performed using the Chi-squared or Fisher’s exact test, as appropriate. Continuous variables were presented as the mean ± 1SD for parameters with normal distribution and as the median [25–75%] for parameters with non-normal distribution. Between-groups comparison were performed using two-tailed, unpaired t-tests or the Mann–Whitney U test for parameters with non-normal distribution. Urinary albumin (mg/gCr) was also interpreted with Log urinary albumin (geometric mean ± SD). Univariable and multivariable Cox proportional-hazards regression models were used to identify the predictors of long-term MACCE. Variables with a *P*-value < 0.1 on the univariate analysis were entered into the multivariable analysis. Multivariable analysis was performed using stepwise selection methods. The cumulative probability of events was estimated using Kaplan–Meier analysis. All *P*-values < 0.05 were considered statistically significant. Statistical analyses were performed using JMP Pro version 16 software (SAS Institute Inc., Cary, North Carolina, USA; https://www.jmp.com/en_us/home.html).

### Ethics approval and consent to participate

All participants provided written informed consent before enrolment in the present study, which was conducted in accordance with the Declaration of Helsinki and approved by the local ethics committee.

### Consent for publication

All authors consent to this publication.

## Results

**Figure 1:**
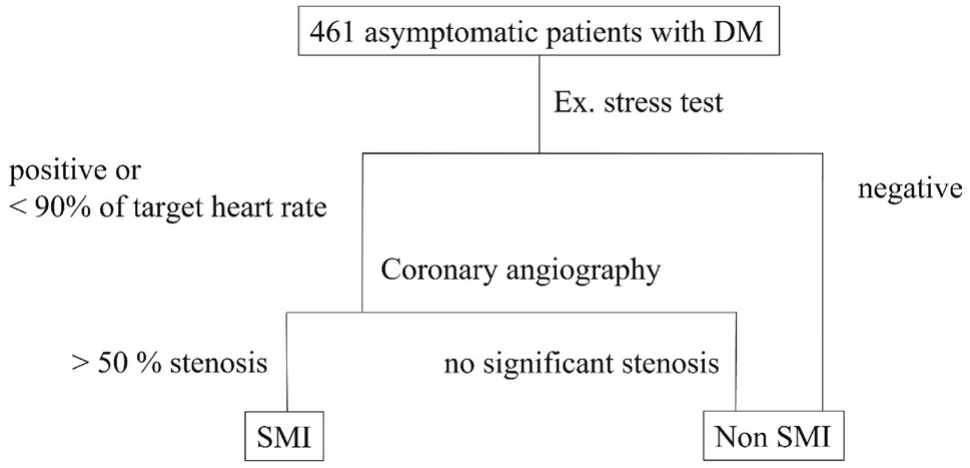
Study protocol. A total of 461 consecutive patients with diabetes mellitus were prospectively studied between July 2011 and August 2017. All patients were asymptomatic and self-sufficient in daily life and underwent the ERG test. Coronary angiography was performed if the stress test was positive, or if the patients did not achieve 90% of the target heart rate. Non-SMI was diagnosed without coronary angiography if the ERG was negative. Thirty-eight patients underwent coronary angiography despite a negative exercise test. None of the patients had a significant coronary lesion. *ERG* ergometer exercise, *SMI* silent myocardial ischaemia.

This study enrolled 461 consecutive outpatients with DM between July 2011 and August 2017. The mean age of the study cohort was 61 ± 15 years; 291 patients were men and 170 patients were women. The average disease duration was 7.9 ± 8.4 years. Figure 1 depicts the flowchart of the study enrolment. A total of 134 patients showed a positive ECG result or were unable to complete the exercise test due to fatigue or dyspnea. A total of 81 patients were diagnosed with SMI based on the exercise stress test and coronary angiography, according to the protocol. Thirty-eight volunteers underwent coronary angiography despite a negative exercise test. None of the patients had a significant coronary lesion.

The patients in the SMI group were older (69.0 ± 12.0 vs. 59.2 ± 14.7 years, *P* < 0.001), with a longer disease duration of DM (11.6 ± 9.1 vs. 7.1 ± 8.0 years, *P* < 0.001), and had a higher incidence of retinopathy (38.3 vs. 18.0%, *P* < 0.001) and prior myocardial infarction (19.8 vs. 4.7%, *P* < 0.001) compared to the non-SMI group. (Table 1) Moreover, the eGFR was significantly lower in the SMI group than that in the non-SMI group (70.5 ± 23.8 vs. 81.8 ± 30.0 mL/min/1.73 m^2^, *P* < 0.001). The HbA1c level and proteinuria were similar between the two groups.

**Table 1:** Baseline Clinical Characteristics between SMI vs. non SMI in DM.

Variable	SMI	non SMI	*P* value
	n = 81	n = 380	
Age (years)	69.0 ± 12.0	59.2 ± 14.7	< 0.001
Female	34 (42.0)	136 (35.8)	0.29
Body mass index (kg/m^2^)	24.0 ± 4.4	25.5 ± 5.5	0.0078
Duration since diagnosis of DM (years)	11.6 ± 9.1	7.1 ± 8.0	< 0.001
Insulin use	28 (34.6)	76 (20)	0.0044
HbA1c (%)	8.6 ± 2.3	8.7 ± 2.6	0.69
Retinopathy	31 (38.3)	68 (18.0)	< 0.001
Hypertension	55 (67.9)	197 (51.8)	0.0084
Dyslipidemia	48 (59.3)	197 (51.8)	0.22
Hyperuricemia	13 (16.0)	42 (11.1)	0.21
Smoking	51 (63.0)	223 (58.7)	0.48
Prior myocardial infarction	16 (19.8)	18 (4.7)	< 0.001
Statin use	25 (30.9)	107 (28.2)	0.62
Serum Creatinine (mg/dl)	0.91 ± 0.88	0.72 ± 0.39	0.2
eGFR (ml/min/1.73 m^2^)	70.5 ± 23.8	81.8 ± 30.0	< 0.001
Proteinuria	29 (35.8)	118 (31.1)	0.4
Urinary albumin (mg/gCr) (n = 344)	62 (23–195)	30 (14–71)	< 0.001
Log urinary albumin (n = 344)	1.85 ± 0.70	1.47 ± 0.59	< 0.001
BNP (pg/ml) (n = 313)	29 (13–73)	14 (7–30)	< 0.001
LVEF (%) (n = 346)	72 (63–75)	72 (68–76)	0.056

Data are expressed as n (%), the mean ± standard deviation (SD) or median (interquartile range); eGFR, estimated glomerular filtration; BNP B-type natriuretic peptide; LVEF left ventricular ejection fraction.

**Figure 2:**
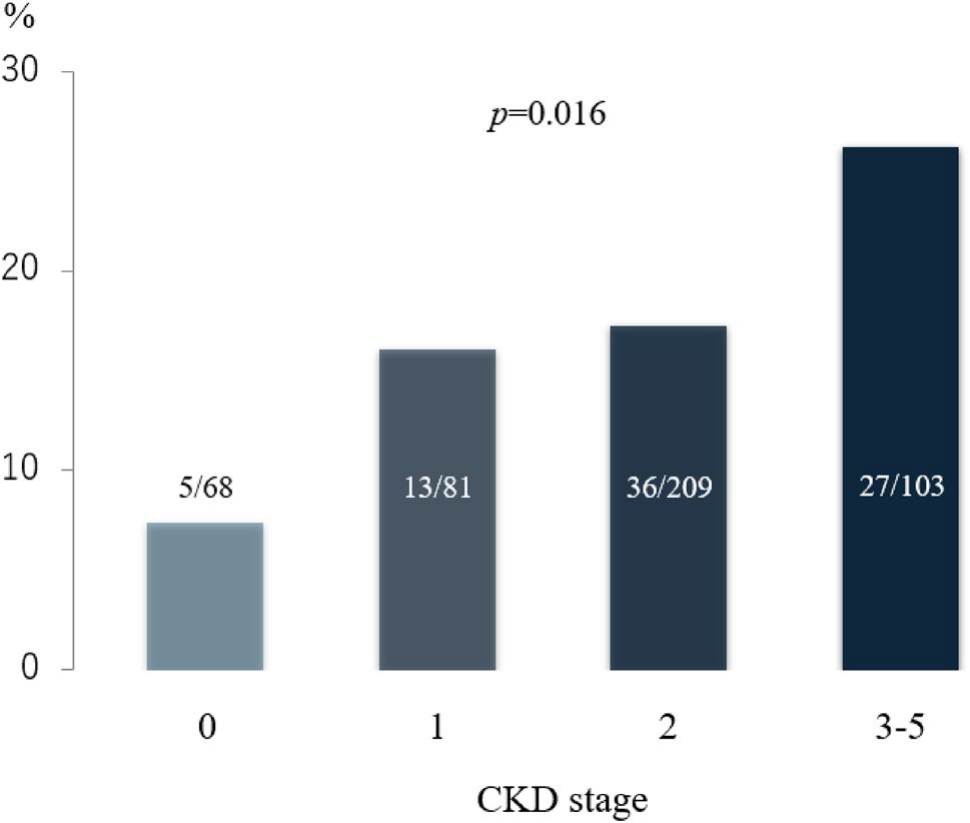
The prevalence rate of SMI according to CKD stage. The prevalence rate of SMI was significantly higher in patients with advanced CKD. *SMI* silent myocardial ischaemia, *CKD* chronic kidney disease.

**Figure 3:**
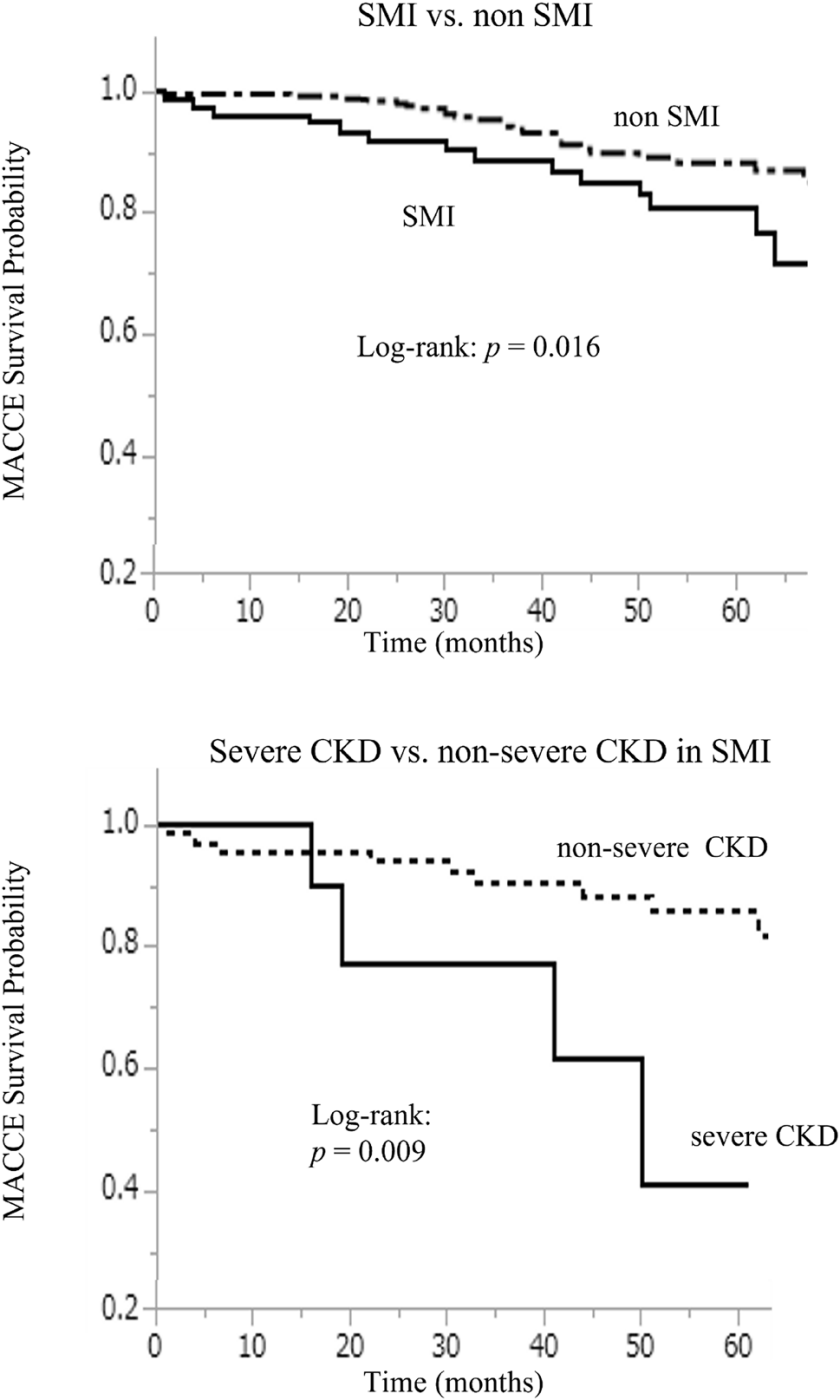
The prognosis of SMI. The median follow-up duration after diagnosis was 35 (15–57) months in the entire cohort of 461 patients with diabetes. The clinical outcomes were poorer in patients with SMI. The severe CKD subgroup (> stage 3a) had a poorer prognosis than that of the non-severe CKD subgroup among the patients with SMI. *SMI* silent myocardial ischaemia, *CKD* chronic kidney disease, *MACCE* major adverse cardiac and cerebrovascular events.

The prevalence of SMI was higher in patients with CKD and the incidence was proportional to the stage of CKD (Fig. 2). SMI occurred more frequently in patients with advanced CKD [27/103 (26.2%) in CKD stages 3–5], whereas only 5/68 patients (7.3%) without CKD, i.e. stage 0, 13/81 (16.0%) with CKD stage 1 and 36/209 (17.2%) in CKD stage 2 had SMI. The median follow-up duration after study enrolment was 35 (15–57) months in the whole cohort of 461 patients. After enrolment, 41 patients with SMI underwent treatment with percutaneous coronary intervention, 4 patients underwent bypass graft surgery, and only optimal pharmaco-therapy was administered to 36 patients. There were 12 all-cause deaths, 3 cases of non-fatal myocardial infarction, and 6 stroke events during the follow-up. The incidence of MACCE was similar between the revascularization group (n = 45) and optimal pharmaco-therapy alone group (n = 36) [8 events (17.8%) vs. 7 events (19.4%), *P* = 0.85]. According to the Kaplan–Meier analysis, the clinical outcomes of the SMI group were poorer than those of the non-SMI group (log-rank: *P* = 0.016) (Fig. 2). The Kaplan–Meier curves revealed that the incidence of MACCE was higher in patients with severe CKD, i.e. > stage 3a, (log-rank: *P* = 0.009) in the SMI subgroup (Fig. 3). There were two patients with dialysis in the present study. One case did not have SMI and died with intracranial bleeding at 72-month after enrollment for the registry. Another case had SMI and underwent coronary bypass graft surgery after diagnosis at enrollment. After that, the case did not have the cardiovascular event such as all-cause death, non-fatal MI and stroke during 48 months.

Urinary albumin (mg/gCr) was independently associated with MACCE in patients with diabetes and SMI [HR 3.37, 95%CI (1.170–9.521), *P* = 0.025], after adjusting for age, sex, and the conventional risk factors presented in Tables 1, 2.

**Table 2:** Multivariable Predictors of MACCE in Diabetic Patients with SMI.

	Hazards ratio	95%CI		*P* value
Age (years)	1.22	1.085	1.443	< 0.001
Log urinary albumin (mg/gCr)	3.37	1.170	9.521	0.025

## Discussion

In the present study we determined the impact of CKD on the incidence of potential myocardial ischaemia and its long-term outcomes in asymptomatic patients with DM. The principal findings of the study were as follows: (1) the prevalence of SMI was higher in the CKD group and its incidence was proportional to the CKD stage, (2) patients with SMI and severe CKD had poor long-term outcomes, and (3) urinary albumin (mg/gCr) was an independent predictor of poor prognosis in diabetic patients with SMI.

The early diagnosis of CAD and optimal treatment according to the guidelines are crucial in specific susceptible populations among asymptomatic patients with diabetes. To our best knowledge, this is the first study to indicate the potential high-risk patients including diagnosis of SMI and long-term outcomes. The result of the present study demonstrates that CKD is a key factor during the examination and management of SMI in asymptomatic patients with DM in routine clinical practice.

### Previous studies

As mentioned in the Introduction, the subset of asymptomatic patients with DM who are at a higher risk for SMI and cardiovascular events should be identified. There is some evidence for the following factors as predictors of SMI: positive family history of cardiovascular disease, long-standing diabetes, a decreased heart rate response in response to the Valsalva manoeuvre, high level of cholesterol/high-density lipoprotein ratio, high level of fibrinogen, N-terminal fragment of pro-BNP, microalbuminuria and abnormal Holter monitor results^3,11–16^. It is unknown whether those factors are associated with worse long-term outcomes in diabetic patients with SMI^4^. Based on the literature search of PubMed and Cochrane library, the present study is the first to determine the impact of CKD on the incidence of SMI and long-term outcomes in asymptomatic patients with DM.

### Relationship between CKD and SMI

Studies have reported the association of CKD with all-cause and cardiovascular mortality in cohorts comprising the general population^17,18^. In the recent study, an eGFR reduction to less than 60 ml/min/1.73 m^2^ or even a small increase in albuminuria levels was associated with cardiovascular events both in general population and patients with established CKD^19^. Furthermore, albuminuria is known to be associated with cardiovascular events, irrespective of DM^20^, although the underlying pathomechanism is not fully understood. The results of the present study imply that CKD is associated with potential CAD and the prognosis is poor in these patients. Recently, Farag et al. reported that silent myocardial infarction was more common rather than clinical myocardial infarction in asymptomatic patients with advanced CKD. The prevalence of silent myocardial infarction was associated with increased risk of cardiovascular events in the study.　Although the silent or clinical myocardial infarction is different from SMI and the population seemed to be sicker, those findings support the results of the present study^21^.

A previous study demonstrated that the presence of microalbuminuria is a marker of vascular endothelial dysfunction and atherosclerosis^22^. Albuminuria is a marker of structural changes in the renal glomeruli resulting from the collapse in the autoregulation of the glomerular filtration pressure and glomerular endothelial cell dysfunction originally caused by diabetes, hypertension, obesity, and smoking. Moreover, advanced renal dysfunction is the terminal point of glomerular structural change. In a previous study, the presence of albuminuria was associated with impaired flow-mediated vasodilation, representing an estimate of endothelial NO synthesis, irrespective of the presence of diabetes^23^. Thus, the occurrence of albuminuria and reduction in the eGFR may be markers of generalized systemic endothelial dysfunction. It could also offer an explanation for the results of the present study, i.e. CKD is a marker of SMI, while the prognosis was poorer in patients with SMI and severe CKD. An early introduction of drugs intervening such as the renin–angiotensin–aldosterone-system, sodium-glucose cotransporter 2 inhibitors and statin may reduce cardiovascular event risks in CKD patients^24^.

### How to identify and manage SMI in patients with diabetes?

Some non-invasive or less invasive examinations do not require contrast media that can be used to identify SMI in clinical practice such as the exercise test, dobutamine stress echocardiography, or myocardial perfusion single-photon emission CT. Coronary angiography with contrast media is necessary to determine the diagnosis of SMI; however, coronary angiography is seldom performed in patients with CKD due to concerns regarding the incidence of contrast-associated acute kidney injury. It is well established that patients with CKD in whom coronary angiography was avoided exhibited a poorer prognosis^25^. SMI was more prevalent in patients with CKD, who exhibited poor long-term outcomes in the present study. Thus, CKD is the very factor that should be considered for screening CAD. Although the guidelines do not recommend routine examinations for CAD for asymptomatic patients with diabetes, simple examinations, such as ERG test and subsequent coronary angiography, are reasonable for specific populations such as CKD.

The recent ISCHEMIA-CKD trial demonstrated that an initially invasive strategy did not improve the clinical outcomes in patients with moderate or severe ischaemic heart disease and advanced CKD compared to an initially conservative strategy^26^. Approximately half of the patients with SMI underwent revascularisation in the present study. Considering the results of ISCHEMIA-CKD, a more stringent decision for the indications of revascularisation in diabetic patients with SMI may be necessary. In any case, the diagnosis of SMI and the introduction of guideline-directed pharmacotherapy should be necessary to improve the clinical outcomes irrespective of revascularisation.

### Study limitations

There were several limitations to the present study. First, the registry was developed at a single centre. The number of cardiovascular events was small because the study population consisted of outpatients who were asymptomatic, self-sufficient in the activities of daily living, and without frailty. Second, according to the study protocol, patients who tested positive on the ECG stress test were scheduled to undergo coronary angiography. Less invasive examinations such as cardiac perfusion or coronary computed tomography imaging may be more common. Thus, the application of a more conservative diagnostic algorithm could have yielded different results. Finally, the optimal treatment for patients with SMI in diabetes is unclear because randomisation of the treatment strategy for myocardial ischaemia was not implemented. Further studies are needed to determine the optimal treatment for patients with diabetes and CKD and SMI.

## Conclusions

SMI was more prevalent in the CKD group and the incidence was proportional to the CKD stage in asymptomatic patients with DM. The clinical outcomes were poorer in patients with CKD and SMI. CKD may be a key factor during the examination and management of SMI in asymptomatic patients with DM in routine clinical practice.

## Data Availability

The datasets associated with the current study are not publicly available but is provided by the corresponding author on reasonable request only.

## Abbreviations

BNP: Brain natriuretic peptide
CAD: Coronary artery disease
CKD: Chronic kidney disease
CTCA: Computed tomography coronary angiography
DM: Diabetes mellitus
eGFR: Estimated glomerular filtration rate
HbA1c: Haemoglobin A1c
SMI: Silent myocardial ischaemia
ERG: Ergometer exercise
